# Estimating the cost of wounds for the United Kingdom and its countries

**DOI:** 10.1111/iwj.14616

**Published:** 2023-12-27

**Authors:** Keith Harding, Douglas Queen

**Affiliations:** ^1^ Editor‐in‐Chief, IWJ; ^2^ Editor, IWJ

Capturing and understanding the costs of managing wounds within a healthcare facility is hard enough, but doing this for a whole country is next to impossible. Over the years several groups have captured costs within the United Kingdom and have estimated the total cost nationally.^1^, ^2^, ^3^, ^4^, ^5^, ^6^, ^7^, ^8^, ^9^ The authors of these studies detail their limitations and often state that their figures are likely an underestimate of the real cost.

A recent editorial in the International Wound Journal^10^ introduced an approach to estimate the possible costs of wound care using freely available governmental health data,^11^, ^12^ population statistics^13^ and the research findings of many national groups,^1^, ^2^, ^3^, ^4^, ^5^, ^6^, ^7^, ^8^, ^9^ within the United Kingdom. Using the methodology of Queen and Harding,^10^ an estimate of the costs of wounds in both the United Kingdom as a whole, and the individual countries of which it is comprised was carried out.

The following figure provides a snapshot of the possible costs of wound care within the United Kingdom in the year 2022.

From the previous editorial^10^ it was estimated that the costs of wound care in the United Kingdom (2019) were 10.1 billion PPP International Dollars (or 6.8 billion GBP—using the IMF Conversion Rate^14^). An international dollar being defined as being able to buy in the cited country a comparable amount of goods and services a US dollar would buy in the United States.^15^ Our analysis shows a significant increase over the 3 years since the original editorial.^10^

**FIGURE 1:**
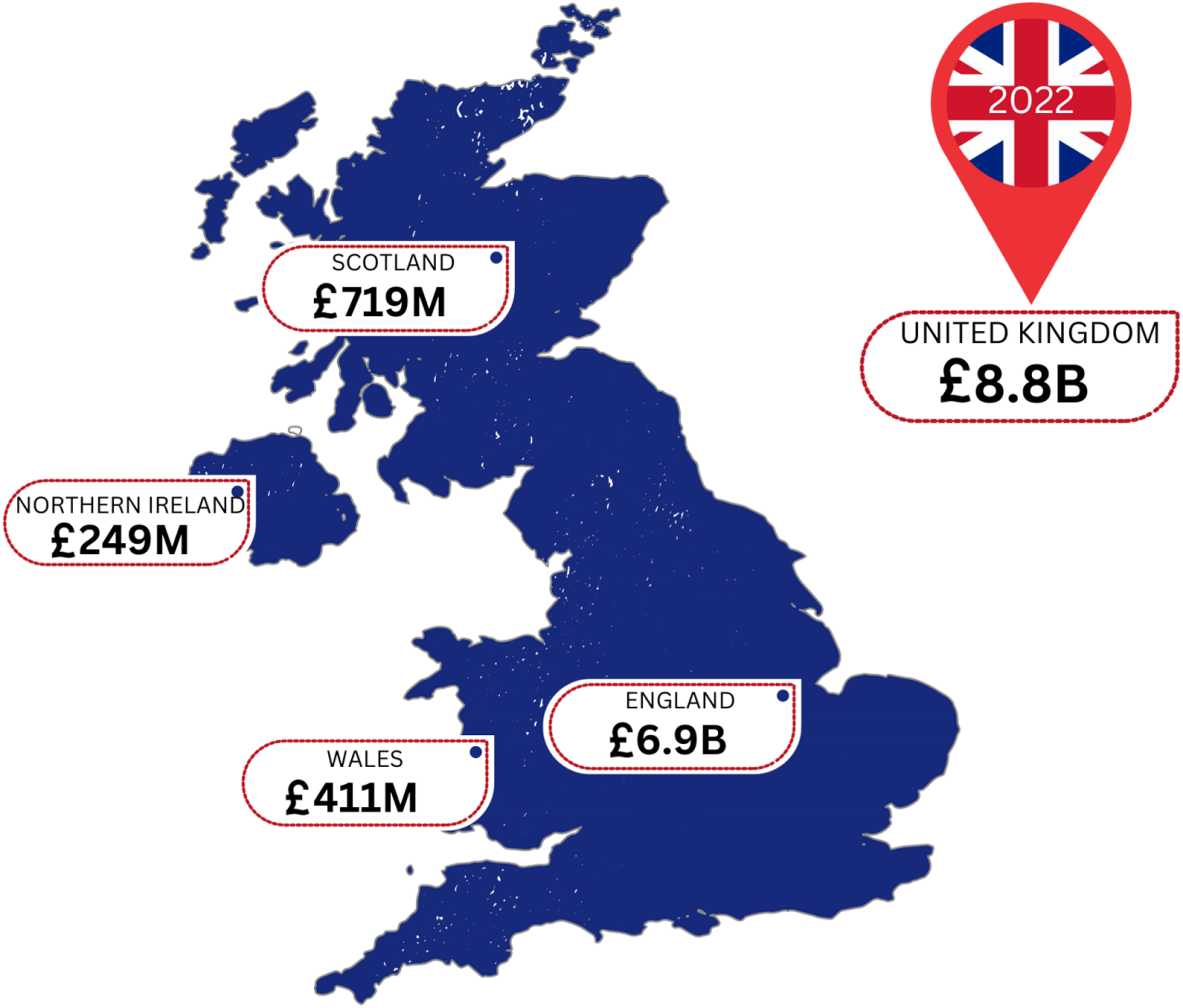
Estimated costs of wound care within the United Kingdom.

As discussed in a recent editorial on Canadian costs, the authors detailed the potential impact of COVID‐19 in the subsequent years but regarded this as not impacting the likely estimate of costs.^16^, ^17^ The data presented in Figure 1 provides a crucial estimate of the likely costs of wounds across the United Kingdom. The costs are significant across all geographies of the United Kingdom and the breakdown aligns with previous studies in some of these geographies.^5^, ^6^ These figures can provide a vital benchmark with regard to governmental impacts both regionally and nationally.

Comprehending the economic impact of wound care offers valuable insights to policymakers and healthcare leaders, shedding light on the broader economic implications of wound management and its costs to the National Health Service. This knowledge serves as a foundation for informed decision‐making and the development of policies and research direction that support effective wound prevention and care practices.

While the IWJ has pledged to update the global picture annually,^10^ including the United Kingdom, we encourage national UK entities to provide the regional updates regularly to keep researchers up to date with the most recent estimates based on updated government statistics and any local research findings.
